# Mechanical Behavior of Anchor Bolts for Shallow Super-Large-Span Tunnels in Weak Rock Mass

**DOI:** 10.3390/ma16175862

**Published:** 2023-08-27

**Authors:** Shaohui He, Jiaxin He, Jianfei Ma, Xiabing Liu, Yiming Li, Bin Zhang

**Affiliations:** 1 School of Civil Engineering, Beijing Jiaotong University, Haidian District, Beijing 100044, China; jiaxin_he@bjtu.edu.cn (J.H.); majfncut@163.com (J.M.); 19115028@bjtu.edu.cn (Y.L.); 21115026@bjtu.edu.cn (B.Z.); 2 Guangdong Hua-Lu Transport Technology Co., Ltd., Guangzhou 510420, China; liuxb_1989@163.com

**Keywords:** shallow super-large-span tunnels, anchor bolts, mechanical behavior, weak rock mass, numerical simulation, field monitoring

## Abstract

Based on the Xiabeishan No.2 tunnel project of the Hang-Shao-Tai high-speed railway in China, the mechanical behavior of the anchor bolts for shallow super-large-span (SSLS) tunnels in weak rock mass is comprehensively investigated through laboratory tests, numerical simulation, and field tests. Firstly, an eight-month field test is conducted in the Xiabeishan No.2 tunnel, and it is discovered that the blasting vibration created by the construction of the middle pilot tunnel caused serious damage to the temporary support, seriously affecting the development of the bolt axial force and causing great construction risks. Then, the refined finite difference model of the SSLS tunnels is formulated, and a series of field and laboratory tests are conducted to acquire the calculation parameters. By comparing the monitored and simulated bolt axial force, the reliability of the numerical model is verified. Subsequently, the influence of the rock condition, construction scheme and bolt length on the mechanical behavior of anchor bolts is discussed. It is revealed that the rock grade significantly affects the bearing characteristics of anchor bolts. The construction scheme can greatly affect the magnitude and development mode of the bolt axial force, but the final distribution characteristics of the bolt axial force do not change regardless of the construction sequence. The axial force of the anchor bolts grows rapidly with the bolt length when the bolt length is within 18 m; meanwhile, when the bolt length exceeds 18 m, increasing the bolt length has a limited effect on the improvement in the bolt support performance. Finally, some optimization measures are proposed according to the monitoring data and simulation results.

## 1. Introduction

In recent years, super-large-span tunnels, with their spaciousness and higher traffic efficiency, have been recorded all around the world; some examples include the Tomei-Meishin highway tunnel in Japan [1,2], the Sapaesan tunnel in Korea [3], the Pinheiros tunnel in Brazil [4], the Liantang tunnel [5], and the Badaling Great Wall Station in China [6], with certain tunnels reaching spans greater than 25 m. However, the increasing excavation area and tunnel span of super-large-span tunnels have also brought serious challenges to construction, operation, and maintenance [7,8,9]. In particular, in the weak rock stratum, if the designed support parameters do not meet the deformation requirement, severe safety accidents may occur [10,11]. In addition, as the most widely used excavation method in super-large-span tunnels, the sequential excavation method could ensure excavation safety, but the construction efficiencies are not well optimized [12,13]. What is more, due to the numerous pilot tunnels and complicated construction steps required, namely, the dynamic construction model, it is challenging to evaluate the mechanical characteristics and stress distribution of support structures. Thus, optimized designs for the construction scheme and support structures of super-large-span tunnels are of great importance.

With the rapid development of the New Austrian Tunnelling Method [14,15], the anchor bolt–steel mesh–shotcrete (ASS) system has been widely adopted in underground structures, such as mountain tunnels and mining engineering [14,16]. As a result, the anchorage effect and new-style support have attracted the attention of more and more scholars [17,18,19,20]. For example, Zhang et al. [21] explored the anchorage performance of anchor bolts in the bedded rock mass, and examined the failure mode under tensile and shear stress. Based on the bolt–rock interaction model, Zhao et al. [22] derived a theoretical solution for the internal force of anchor bolts, and then proposed an anchor–rock interaction model of the fully grouted anchor bolts. By utilizing laboratory tests and theoretical analysis, Zhao et al. [23] investigated the deformation and axial force of rock bolts, anchored bodies, and rock mass under shear action. Taking the Muzhailing tunnel as the research context, the mechanical responses of anchor plates with different shapes and sizes have been systematically explored in squeezing large-deformation tunnels [24]. Zhao et al. [25] put forward an anchor bolt developed using a basalt-fiber-reinforced polymer (BFRP), and the corresponding structure parameters and anchorage lengths were examined through numerical simulation and laboratory tests.

The aforementioned studies have produced scientific guidance for the optimization of the construction reinforcement and support structures for underground engineering in the context of a precipitous topography and complicated geological conditions. However, the tunnel spans in the above studies are relatively small (less than 18 m). For super-large-span tunnels with a span greater than 25 m, the interactions among their huge size, poor geological conditions, and complicated construction methods are certain to aggravate the complexity of their stress distributions and mechanical response, and the application of the support structure needs to be further studied. In addition, conflicts regarding anchor bolts have existed for a long time. On the one hand, it is traditionally believed that anchor bolts could effectively strengthen the rock mass or surrounding rocks, and then enhance the construction stability of underground facilities [26,27]. Nevertheless, some scholars believe that the supportive effects of anchor bolts, especially those of systematic anchor bolts, are much weaker under some specific conditions (such as tunnels with high-water-content soil, tunnels with large sections of loess, and soft-rock tunnels), and even advocate removing anchor bolts [28,29].

In view of this, the mechanical behavior of anchor bolts for shallow super-large-span (SSLS) tunnels (the span is 26.3~27.3 m) in weak rock mass is comprehensively investigated in this paper. Firstly, an eight-month field test is performed in the Xiabeishan No.2 tunnel to investigate the development and distribution characteristics of bolt axial force under different surrounding rock conditions. Then, the refined finite difference model of the SSLS tunnels is formulated, and a series of field and laboratory tests are conducted to acquire the calculation parameters. By comparing the monitored and simulated bolt axial force, the reliability of the numerical model is verified. Subsequently, the influence of the rock condition, construction scheme and bolt length on the mechanical behavior of anchor bolts is discussed. Finally, some optimization measures are proposed according to the monitoring data and simulation results. The research findings could provide a reference for the bolt design of SSLS tunnels.

## 2. Research Background

### 2.1. Prototype Tunnel

**Figure 1:**
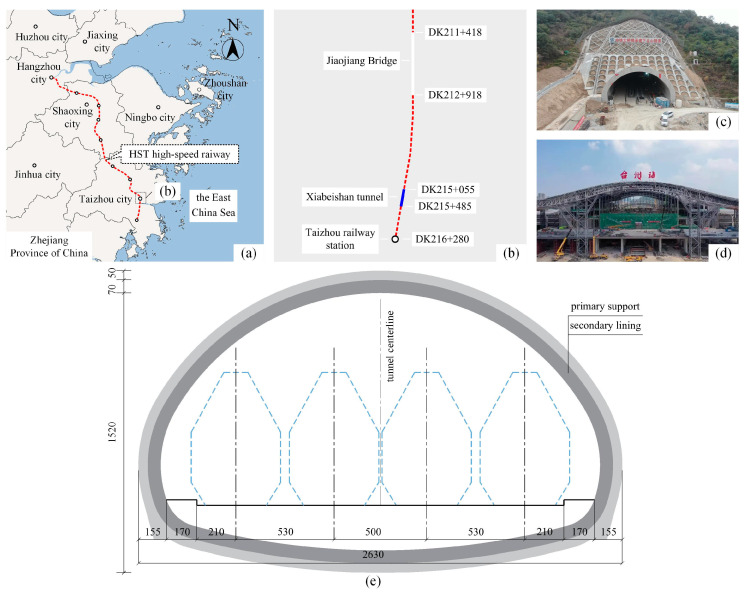
Project overview; (**a**) project location; (**b**) tunnel surroundings; (**c**) Xiabeishan No.2 tunnel; (**d**) Taizhou railway station; (**e**) tunnel cross-section (units: cm).

As shown in Figure 1, the Hang-Shao-Tai high-speed railway was designed to facilitate the traffic and economic development of the cities of Hangzhou, Shaoxing, and Taizhou in China. The Xiabeishan No.2 tunnel, located in Taizhou, was designed as a four-line shallow super-large-span (SSLS) tunnel since it is close to Taizhou railway station (only 795 m away). The tunnel’s general span is 26.3 m, and its maximum span is 27.3 m. The length, maximum depth, and maximum excavation area of the tunnel are 414 m, 57.4 m, and 361.0 m^2^, respectively.

### 2.2. Geological Conditions and Construction Parameters

**Figure 2:**
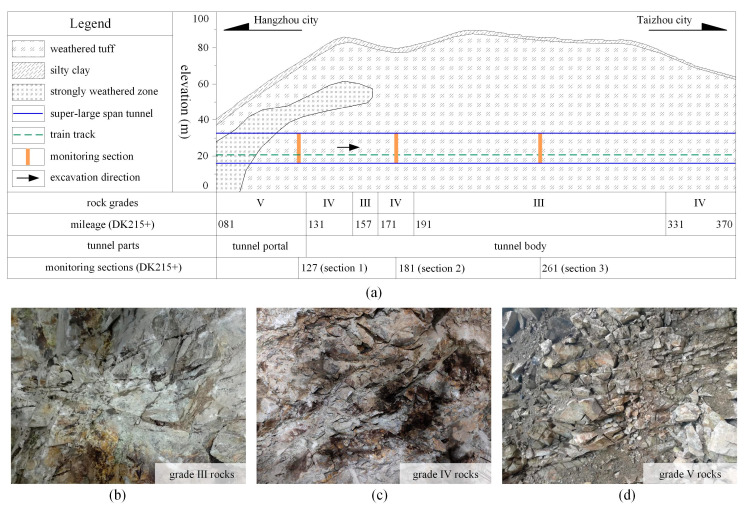
Geological conditions; (**a**) longitudinal section; (**b**) grade III rock mass; (**c**) grade IV rock mass; (**d**) grade V rock mass.

The geological condition diagram of the Xiabeishan No.2 tunnel is plotted in Figure 2. The mountain surface is covered with 2~4 m of thick eluvium (i.e., silty clay), and the inside is weathered tuff. Owing to the strong weathering effect, the joints of rock mass at the tunnel portal section are extremely developed, with 2~30 cm diameter rock blocks and a 1~5 cm thick clay layer between the joint fissures. Overall, the weathering degree of the rock mass decreases from the tunnel portal section to the tunnel body along the longitudinal direction. Referring to the Chinese classification [30], the rock masses of the tunnel portal section and tunnel body of the Xiabeishan No.2 tunnel are grade V and grades III~IV, respectively.

**Figure 3:**
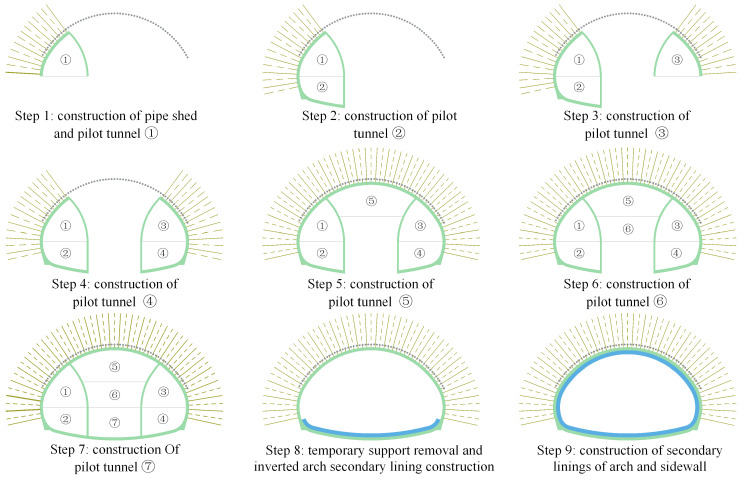
Site construction sequence of the Xiabeishan No.2 tunnel.

**Figure 4:**
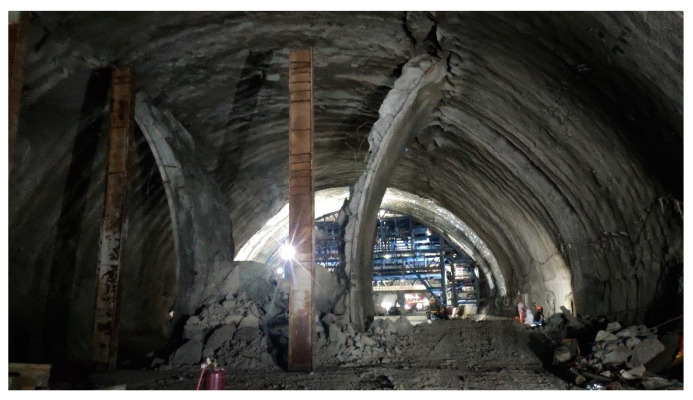
Construction site of the Xiabeishan No.2 tunnel.

The support system of the Xiabeishan No.2 tunnel is listed in Table 1, and the support parameters have been adjusted referring to the rock mass’s properties. As plotted in Figure 3, the double-side heading method, with an excavation sequence from the side pilot tunnels to the middle pilot tunnel, is employed in the present project. The bench lengths of the side pilot tunnels and middle pilot tunnel are 10~20 m and 10 m, respectively, and the lag distance of the adjacent pilot tunnels is 10~20 m. The excavation footages in the grade III, Ⅳ, and V rock mass are 1.2 m, 1.0 m, and 0.8 m, respectively. During the removal of the temporary support, the segmented construction method is implemented to enhance efficiency, and each segment is 10 m. The working face of the temporary support demolition is 10~20 m behind the excavation face. The construction site of the SSLS tunnel is shown in Figure 4.

## 3. Field Test

### 3.1. Monitoring Scheme

**Figure 5:**
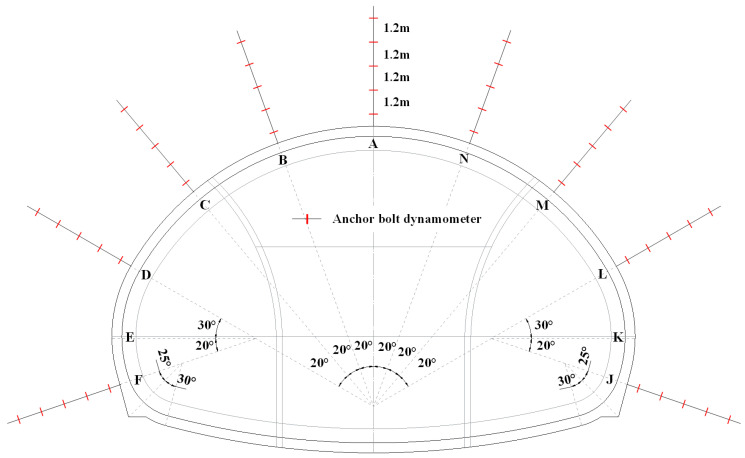
Layout of measuring points in field monitoring.

**Figure 6:**
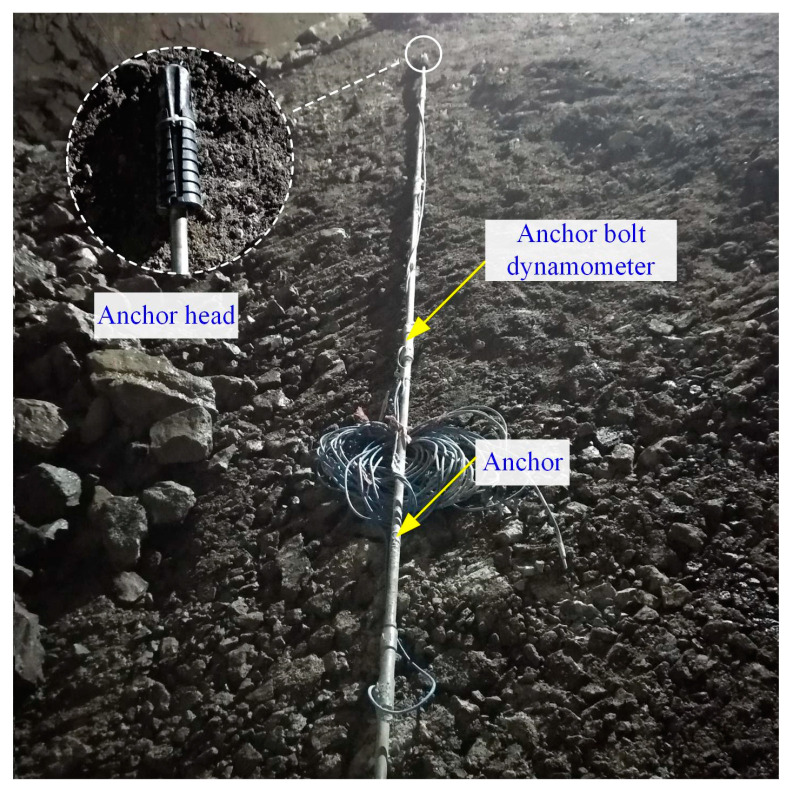
Installation of axial force dynamometer of anchor bolts.

An eight-month field investigation was carried out in the Xiabeishan No.2 tunnel to explore the mechanical characteristics of anchor bolts. Three monitoring sections with a mileage of DK215 + 127, DK215 + 181, and DK215 + 261 were chosen, namely section 1~3, respectively. The surrounding rock masses of section 1~3 were grade V, IV and III, respectively. Nine measuring lines which are located at A-F and J-N for the bolt axial forces were arranged, as plotted in Figure 5. Five dynamometers with an interval of 1.2 m were welded on the measuring lines, as shown in Figure 6.

### 3.2. Monitoring Results and Analysis

**Figure 7:**
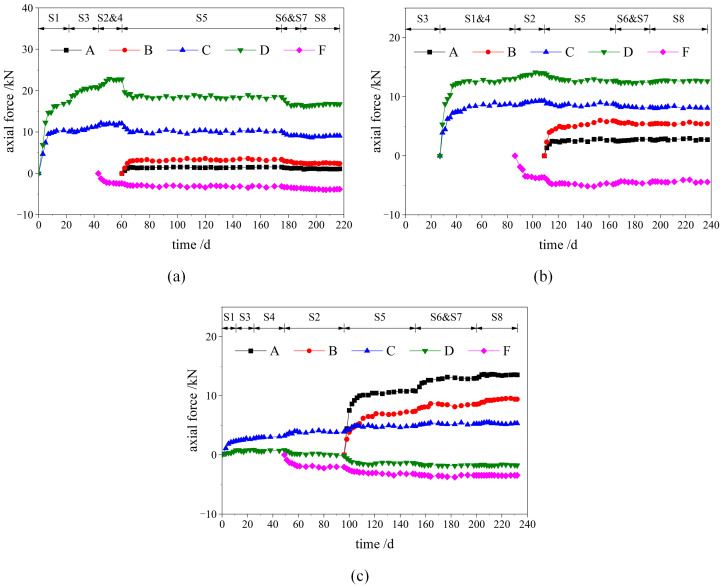
Development of the bolt axial force; (**a**) section 1; (**b**) section 2; (**c**) section 3.

Figure 7 displays the development of the bolt axial force in the SSLS tunnel. For convenience, each anchor bolt only shows the development of the maximum value. In the grades IV and V rock mass, the increase in the bolt axial force in the side pilot tunnels mainly occurred in the first two weeks after the excavation of the two side pilot tunnels (S1~S4). This finding can be attributed to the fact that the stress release of the surrounding rock is mainly concentrated in the early stage of excavation. When the middle pilot tunnel was excavated (S5~S7), the axial force of the anchor bolts in the side pilot tunnels decreased by 15~35%, while the bolt axial force in the middle pilot tunnel remained small. These results are due to the super-large-span structure formed after the middle pilot tunnel’s excavation, and because the overall subsidence occurred in the overlying rock mass under the poor rock mass conditions, which is not conducive to fulfilling the role of bolt support. In the stage of temporary support removal (S8), the bolt axial force at each position of the SSLS tunnel remained almost unchanged. It was found that the blasting vibration created by the construction of the middle pilot tunnel caused serious damage to the temporary support, and the internal force adjustment of the rock mass and supporting structure was completed before the temporary support was demolished, which caused great construction risks. Therefore, the construction management should be strictly strengthened to ensure the accuracy of drilling and charging, and to minimize the damage that the blasting vibration causes to the temporary support. In addition, the reinforcement ratio of the temporary support should be appropriately increased to improve its strength and toughness.

In the grade Ⅲ rock mass, after the construction of the side pilot tunnels (S1~S4), the bolt axial force in the side pilot tunnels was much smaller than that in the grades IV and V rock mass. This result can be attributed to the good rock quality, the fact that the rock deformation induced by the construction of the side pilot tunnels is small, and the bolt support required for the rock mass being relatively weak. Then, the formation of a super-large-span structure in steps S5~S7 increased the demand of the surrounding rock for bolt support. The bolt axial force in the two side pilot tunnels increased by 20~30%, and the largest axial force of the anchor bolts was observed in the middle pilot tunnel. In the stage of temporary support demolition (i.e., Step S8), the bolt axial force at each position of the tunnel changed slightly, which is consistent with the results obtained for the grades IV and V rock mass. This result once again indicates that the internal force adjustment of the surrounding rock and supporting structure was completed before the temporary support demolition.

**Figure 8:**
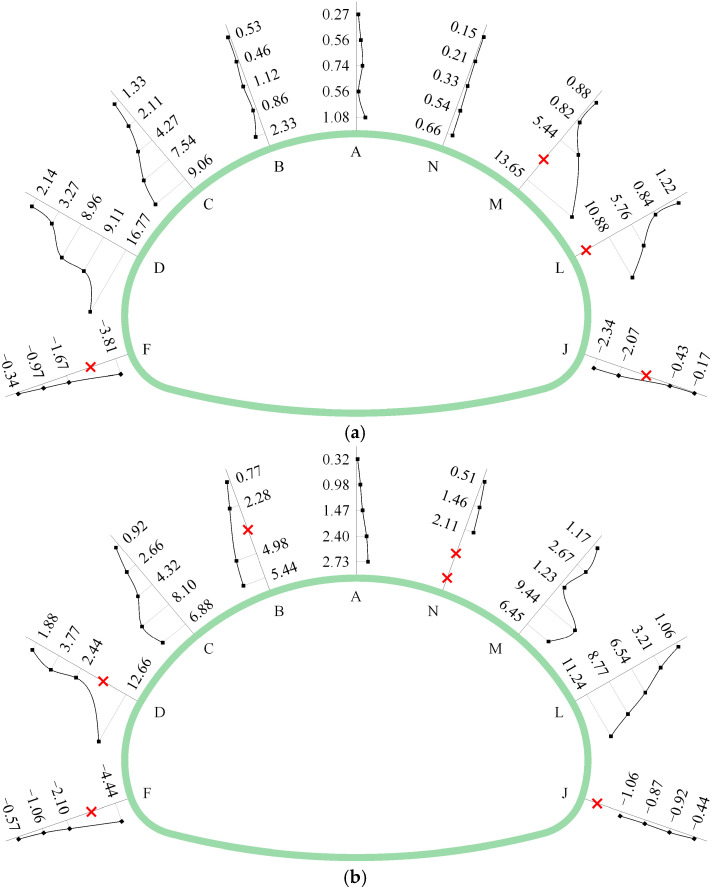

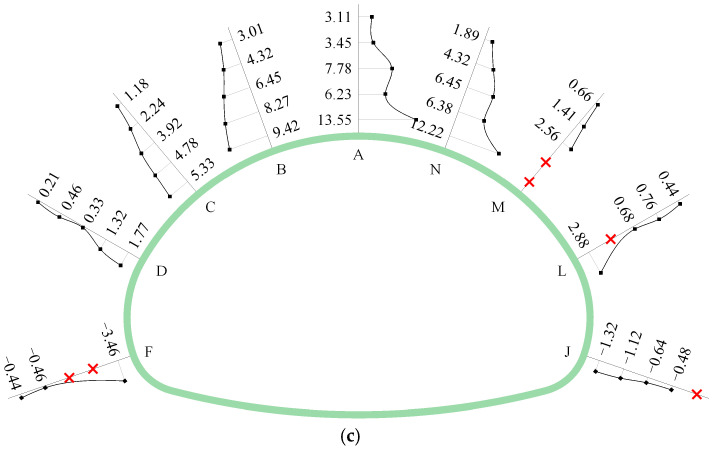
Final distribution of bolt axial force (units: kN); (**a**) section 1; (**b**) section 2; (**c**) section 3; (symbols “×” indicate that the monitoring instrument has been damaged).

Figure 8 shows the final distribution of the bolt axial force of the SSLS tunnel. No matter what the surrounding rock condition is, the axial force of the anchor bolt is always concentrated at the measuring points near the tunnel. With the increase in the radial distance, the axial force of the bolt decreases gradually; this is caused by the concentration of friction between the bolt and the surrounding rock. In the grades IV and V rock mass, the distribution of the bolt axial force is large in the side pilot tunnels and small in the middle pilot tunnel, which implies that the anchor bolts at the side pilot tunnels can provide effective support for the surrounding rock, while those at the middle pilot tunnels are unable to properly act as supports. However, in the grade III rock mass, the distribution pattern of the bolt axial force is large in the arch and small in the sidewall, which indicates that the bolt’s supporting effect in the tunnel arch is far better than that in the sidewall. The difference between the grade III rock mass and the grades IV~V rock mass can be attributed to the different degrees of overall subsidence in the overlying rock mass. In addition, the anchor bolts at the wall foot of the tunnel (i.e., points F and J) are always under pressure in the grades III~V rock mass, and present a limited supporting effect. It is recommended that the anchor bolts are replaced with other reinforcement measures.

## 4. Simulation Method and Materials

### 4.1. Finite Difference Model

**Figure 9:**
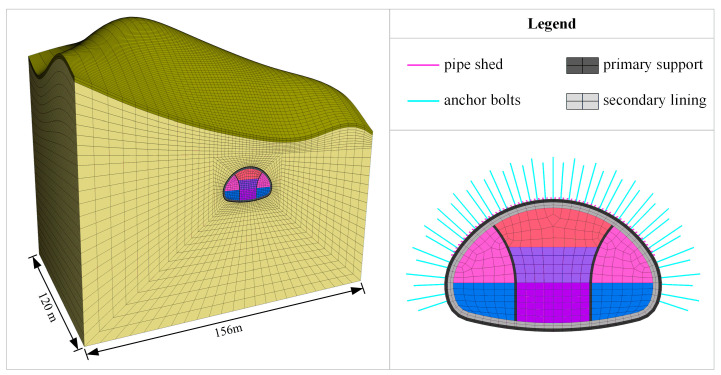
Refined numerical model.

As plotted in Figure 9, the finite difference model of the Xiabeishan No.2 tunnel, including the terrain, is formulated using the FLAC 3D program (Version. 6.0). Five times the tunnel radius is set from the model side to the tunnel sidewalls in order to reduce the boundary effect, and the total width, depth, and height of the model are 156 m, 120 m, and 90~140 m, respectively. Fixed and normal constraints are imposed on the model’s bottom and four sides, respectively, and the top of the model is a free surface. In the current study, the rock mass, temporary support, and secondary lining are simulated with hexahedron elements. The Pile element, Beam element, and Shell element are chosen to substitute for the system anchor bolt, pipe shed, and primary support, respectively. In view of the simple and practical characteristics of the Mohr–Coulomb criterion and the small buried depth of the tunnel (the confining pressure of the rock mass is small), it is assumed that the rock mass follows the Mohr–Coulomb criterion. The Hoek–Brown criterion is preferred when the buried depth of the tunnel is large. In addition, the support structure is assumed to be linearly elastic.

In the cyclic construction of the Xiabeishan No.2 tunnel, the steel arch is immediately erected after the excavation of a new tunnel ring, followed by the drilling and installation of systematic anchor bolts; finally, the concrete is sprayed. Thus, in this numerical simulation, the anchor bolts and the initial lining are applied simultaneously after the tunnel excavation.

### 4.2. Material and Parameters

**Figure 10:**
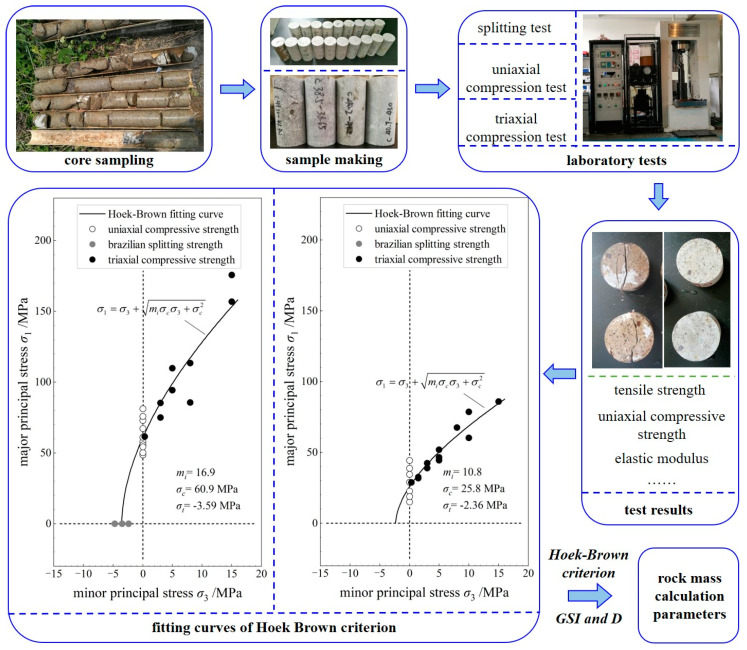
Acquisition of rock mass’s parameters.

As shown in Figure 10, the calculation parameters of the rock mass are derived using field coring, laboratory tests, and geological investigation. Firstly, the tuff samples of Group A (grades III and VI rock mass) and Group B (grade V rock mass) with different weathering degrees are obtained via core-drilling sampling at the tunnel site area. Then, standard cylindrical pieces (50 mm in diameter and 100 mm in height) are prepared according to the Chinese test standard [31]. Later, a series of laboratory tests, including the uniaxial compression test, triaxial compression test, and splitting test, are conducted to collect the mechanical parameters of the tuff samples. The Hoek–Brown nonlinear strength criterion is employed to fit the laboratory test results; the material constant, uniaxial compressive strength, uniaxial tensile strength, and average elastic modulus of the group A samples are 16.9, 60.9 MPa, −3.59 MPa, and 23.4 GPa, respectively, while those of group B are 10.8, 25.8 MPa, −2.36 MPa, and 15.1 GPa, respectively. In addition, the GSI values of the grade III, VI, and V rock masses are determined to be 44, 26, and 12 according to the on-site geological investigation. Finally, the calculation parameters of the grades III, VI, and V rock mass are obtained using the equivalent Mohr–Coulomb shear strength fitting method [32,33,34]. The calculation parameters of the surrounding rock are listed in Table 2. The calculation parameters of the anchor bolts are derived using the pull-out test, as listed in Table 3. The calculation parameters of the pipe sheds are calculated according to their geometric size and are summarized in Table 4.

The mechanical parameters of the steel frames are acquired through the stiffness equivalence method, as calculated in Equation (1).

(1) $$E=E_{c}+E_{s}\frac{S_{s}}{S_{c}}$$

where *E_c_* and *E* represent the elastic modulus of the concrete before and after commutation, respectively. *E_s_* denotes the steel frame’s elastic modulus. *S_c_* and *S_s_* are the cross-sectional area of the concrete and steel frame, respectively. The calculation parameters of the tunnel linings are also listed in Table 2.

### 4.3. Construction Schemes

**Figure 11:**
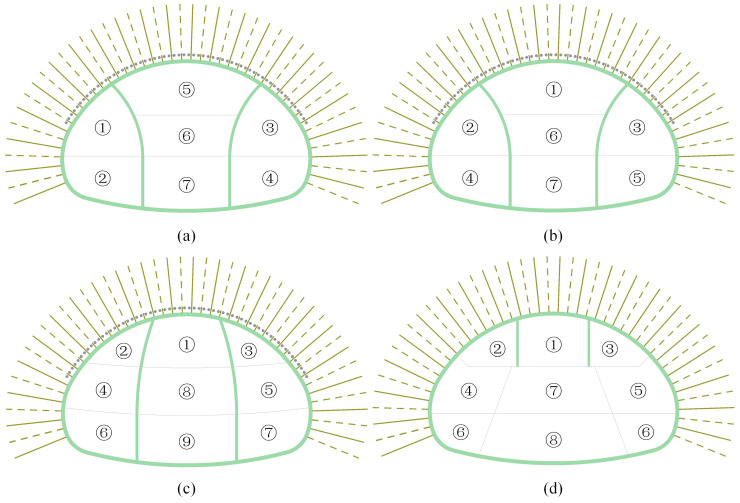
Four construction schemes for SSLS tunnels; (**a**) site construction scheme; (**b**) scheme 1; (**c**) scheme 2; (**d**) scheme 3.

A site construction scheme and three different construction schemes are proposed to investigate the supporting characteristics of the anchor bolts for the SSLS tunnels, as depicted in Figure 11, in which the numbers ①, ②, ③ … represent the excavation sequence of pilot tunnels, and in which the lag distance of the adjacent pilot tunnels is 10 m. According to the excavation sequence of the middle pilot tunnels, the site construction scheme is often referred to as the “side first and then middle” double-side heading method, while scheme 1 and scheme 2 are generally called the “middle first and then side” double-side heading method, and scheme 3 is the three-step method.

### 4.4. Arrangement of Measuring Points in Numerical Simulation

**Figure 12:**
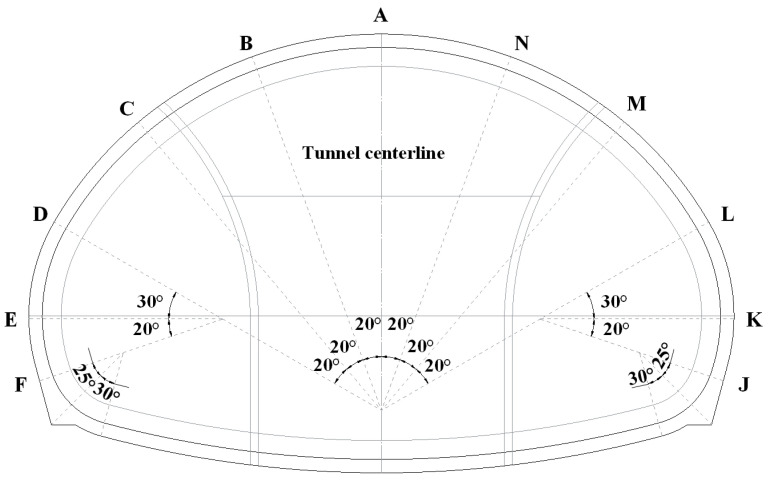
Layout of measuring points in numerical simulation.

Considering the super-large diameter of the Xiabeshan No.2 tunnel, 11 measuring points are arranged along the radial direction, namely, A, B, …, F and J, K, …, N, as shown in Figure 12.

## 5. Simulation Results and Analysis

### 5.1. Comparison between Field Tests and Simulation

**Figure 13:**
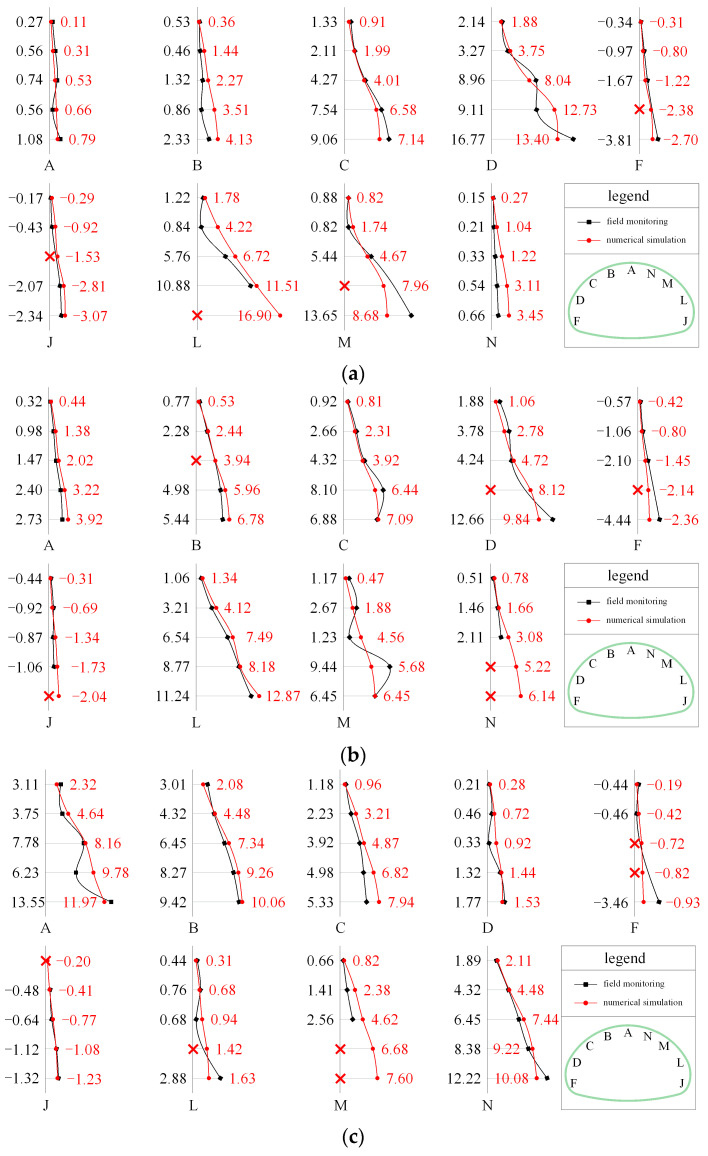
Comparation between numerical simulation and field test (units: kN); (**a**) section 1; (**b**) section 2; (**c**) section 3.

As shown in Figure 13, the distribution of the bolt axial force in the numerical simulation is highly similar to the monitoring results. For a single anchor bolt, the maximum axial force always occurs at the end of the bolt near the tunnel. In addition, with the increase in the radial distance, the axial force of the bolt gradually decreases. For the tunnel cross section, the distribution of the bolt axial force is closely related to the surrounding rock conditions. In the grades IV and V rock mass, the bolt axial force presents a distribution mode of “large in the side pilot tunnels and small in the middle pilot tunnel”, while the distribution of the bolt axial force in the grade III rock mass is completely opposite to the former, showing a pattern of “large in the arch and small in the sidewall”. 

In addition, the magnitude of the bolt axial force during monitoring and simulation is very close. Excluding 16 damaged measuring points, the axial force of the anchor bolts is measured normally at 119 measuring points. There are 95 measuring points (about 80%) with errors within 1 kN, 17 measuring points (about 14.3%) with errors between 1 and 3 kN, and a few measuring points (about 5.5%) with errors exceeding 3 kN. These axial force errors may be caused by errors in the installation of the dynamometer. It is worth mentioning that the measuring points with an axial force error exceeding 1 kN are mainly distributed in C, D, L and M of section 1~2 and in A, B and N of section 3. The average value and relative error of the axial force of the bolts at these positions are listed in Table 5. The relative error of the bolt axial force in most positions is less than 10%, which shows that the simulation results are very close to the monitoring data. The relative error at L of Section 1 reaches 43.2%, which is due to the damage caused to some of the measuring points of the bolt at this position. Generally speaking, the distribution and magnitude of the monitored and simulated bolt axial force are very close. Thus, the numerical simulation is more reliable.

### 5.2. Influence of Rock Mass Conditions

**Figure 14:**
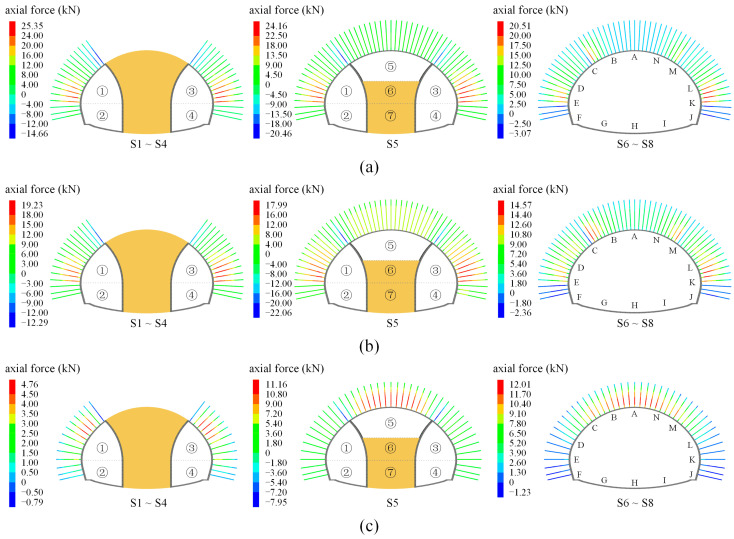
Axial force of anchor bolts under different rock conditions; (**a**) grade V rock mass; (**b**) grade IV rock mass; (**c**) grade III rock mass.

Figure 14 displays the axial force development of anchor bolts for shallow super-large-span (SSLS) tunnels when the site construction scheme is adopted, in which the positive and negative values indicate the tension and compression states, respectively. Firstly, when the two side pilot tunnels are excavated (i.e., Steps S1~S4) in grade IV and V rocks, the anchor bolts in the side tunnels (i.e., points C~E and points M~K) are mainly in tension states. To some extent, the two side pilot tunnels are equivalent to two deep-buried tunnels of a normal size. At this stage, the overall subsidence of the overlying rock mass is weak, which is conducive to fulfilling the role of bolt support. Then, the bolt axial force of points C~E and points M~K reduces as the upper bench of the middle column (i.e., part ⑤) is excavated, and the bolt axial force of the side pilot tunnels is larger than that of the middle pilots. In this construction step, the excavation of part ⑤ connects the side pilot tunnels, forming a super-large flat tunnel. The interaction of the weak rock condition and the super-large-span structure aggravates the overall subsidence of the overlying rock mass; thus, the application of anchor bolts in the middle pilot tunnel presents a limited anchoring effect. In excavation steps S6, S7, and S8, the bolt axial force of the side pilot tunnels reduces by 15~20%, and that of the middle pilot tunnels presents few changes. These phenomena are due to the fact that the removal of the temporary support intensifies the overall subsidence effect of the overlying rock mass, and thus decreases the supportive effect of the anchor bolts. The final distribution of the axial force of the anchor bolts presents a mode of “large in the side pilot tunnels and small in the middle pilot tunnel”, which means that the anchor bolts of the side pilot tunnels in the grades IV and V rocks play a good supporting effect, while those in the middle pilot tunnels display a limited effect.

In grade Ⅲ rock mass, the anchor bolts on the side pilot tunnels are in also a tension state after Steps S1~S4, but the axial forces are far lower than those of grades IV and V rocks. With a better rock quality, the rock subsidence of the grade Ⅲ rock mass is obviously weaker than that of the grades IV and V rocks, and the corresponding axial forces of the bolts are much lower than the weak rock mass. In Step S5, however, the axial force shows the distribution pattern of “large in the arch and small in the sidewall”, which is completely opposite to the distribution of the grades IV and V rock mass. This phenomenon is because the overall subsidence effect of the grade III surrounding rock is relatively weak, which is beneficial to fulfilling the role of bolt support in the middle pilot tunnel. After steps 6~8, the axial force of the anchor bolts at the tunnel arch increases by 10%, while the anchor bolts at the side wall (i.e., points D~F and points L~J) still present small axial forces (close to zero). Therefore, it is advised that the anchor bolts at the sidewalls are removed.

### 5.3. Influence of Construction Schemes

**Figure 15:**
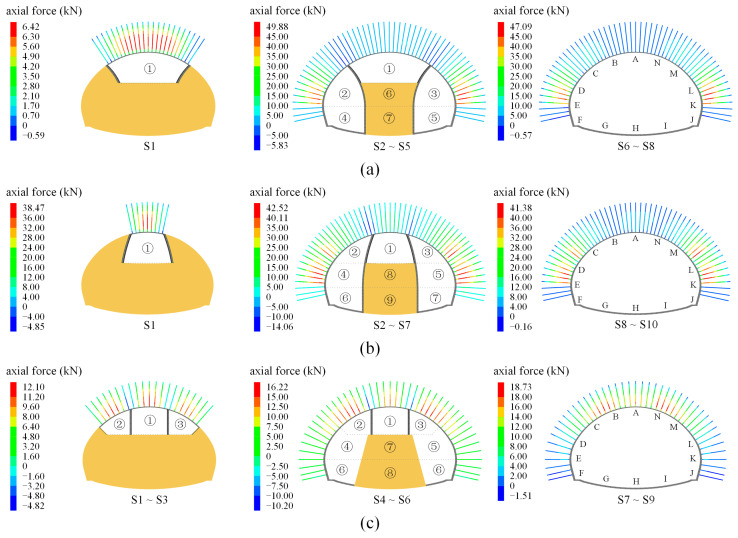
Axial forces of anchor bolts under different construction methods and rock conditions; (**a**) scheme 1 in grade V rock mass; (**b**) scheme 2 in grade V rock mass; (**c**) scheme 3 in grade III rock mass.

Figure 15a,b exhibits the development of the bolt axial forces when schemes 1 and 2 are used in the grade V rock mass. The development of the bolt axial forces of the two schemes is very similar. When the upper bench of the middle pilot tunnel is excavated (i.e., Step S1), the anchor bolts at the tunnel arch are mainly in a tension state, but the tension is relatively small due to the influence of the overall subsidence effect. After the excavation of the two side pilot tunnels, a super-large flat tunnel forms, and the aggravated overall subsidence effect leads to a decrease in the bolt axial force. However, the axial force of the anchor bolts at the side walls is 40~50 kN, which is about twice as much as that of the site construction scheme (as shown in Figure 14a). This result indicates that the supporting effect of the anchor bolts at the side pilot tunnels significantly improves when the middle pilot tunnel is excavated first. Furthermore, when the residual steps are finished, the overall subsidence effect is aggravated again, which leads to a slight decrease in the bolt axial force. At this time, the bolt axial force presents a distribution mode of “large in the side pilot tunnels and small in the middle pilot tunnel”, which is consistent with the site construction scheme. This phenomenon implies that the excavation sequence of the pilot tunnels could affect the magnitude and evolution mode of the bolt axial forces, but that it could not change the final distribution characteristics.

Figure 15c shows the development of the bolt axial force for scheme 3 in the grade Ⅲ rock mass. After the excavation of the upper bench of the SSLS tunnel (i.e., steps ①~③), the maximum tensile force of the bolt at the tunnel arch (i.e., points B~N) is 12.1 kN. Then, with the excavation of the side pilot tunnels, the maximum axial force of the anchor bolts in the tunnel arch increases to 16.2 kN, while that of the sidewalls (i.e., points D~F, and points L~J) is within 4 kN. When the excavation steps S7~S9 are finished, the axial force of the anchor bolts in the tunnel arch increases again, while that in the sidewalls remains small. The bolt axial force presents a distribution of “large in the arch and small in the sidewalls”, which is consistent with the site construction scheme in the grade Ⅲ rock mass (as shown in Figure 14c); however, the axial force values are about 50% more than the latter. This result also shows that the construction method can significantly affect the magnitude and development mode of the bolt axial force, but that the final distribution characteristics of the bolt axial force do not change regardless of the construction sequence. In addition, when scheme 3 is employed, the anchor bolts in the tunnel arch could have a better bearing capacity.

### 5.4. Influence of Bolt Length

**Figure 16:**
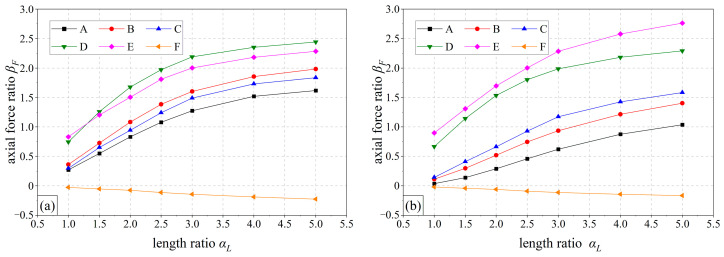
Curves of bolt axial force with bolt length; (**a**) grade IV rock mass; (**b**) grade V rock mass.

In the design of anchor bolts, too short a length will greatly weaken the supporting effect of anchor bolts, while too long a length will dramatically increase the construction cost and cause a lot of waste. Therefore, it is of great engineering value to reasonably adjust the bolt length. Figure 16 shows the relationship between the bolt axial force and the bolt length at various positions of the SSLS tunnel in the grade IV and grade V rock mass. The abscissa $\alpha_{L}$ is the ratio of the bolt length to the initial length *L_i_*, and *L_i_* in the grade IV~V rock mass is 6 m; the ordinate $\beta_{F}$ is the ratio of the bolt axial force to *F_i_*, and *F_i_* is the maximum axial force when the bolt length is the initial length; *F_i_* is 14.6 kN and 20.5 kN in the grade IV and grade V rock mass, respectively. The bolt axial force increases rapidly with the bolt length when α*_L_*≤ 3, and the growth rate slows down when α*_L_ >* 3, which indicates that when the bolt length exceeds three times the initial length (i.e., 18 m), increasing the bolt length has a limited effect on improving the support performance of anchor bolts. Therefore, in the design of anchor bolts for SSLS tunnels, the bolt length can be appropriately increased to improve the supporting effect, but the bolt length should be controlled within 18 m as far as possible to ensure the balance between the supporting effect of the bolt and the engineering economy. However, the anchor bolts at the wall foot (i.e., point F) are always in compression, which indicates that the supporting effect of the bolt at the wall foot is always poor, and that increasing the bolt length has little effect on improving the supporting effect of the bolt at this position. Therefore, the anchor bolts at the wall foot should be cancelled to save the construction cost.

## 6. Bolt Design Optimization

According to the monitoring data and simulation results, the following suggestions for anchor bolts in SSLS tunnels are put forward.

(1) Regardless of the variations in the rock condition, construction method and anchor length, the anchor bolts at the wall foot (points F and J) of the SSLS tunnel are always in compression, which implies that the anchor bolts’ supporting effect at the wall foot is limited. Therefore, it is suggested that the anchor bolts in this part are removed to save costs and improve the construction efficiency.

(2) In grades IV~V rock mass, the anchor bolts in the side pilot tunnels can always play a good supporting role. However, due to the influence of the overall subsidence effect of the overlying rock mass, the anchor bolts in the middle pilot tunnel maintain a small axial force throughout the whole construction process. This may be an effective measure to appropriately increase the bolt length in the middle pilot tunnel so that the anchor head can be fixed in the rock mass, which is less affected by excavation. However, when the bolt length exceeds 18 m, increasing the bolt length has a limited effect on improving the support performance of anchor bolts. In addition, subject to the existing mechanical conditions, an excessive bolt length may lead to the insufficient torque of the drilling machinery. Therefore, the bolt length in the middle pilot tunnel should be controlled within 18 m.

(3) In grade Ⅲ rock mass, the bolt axial force in the sidewall (points D~F and points L~J) is always small. This result is because the horizontal convergence of the surrounding rock in the side wall is very small due to the good rock quality, and the bolt support required for the rock mass is relatively weak. Therefore, in grade Ⅲ rock mass, it is that the anchor bolts at the tunnel side wall are removed to save construction costs.

## 7. Conclusions

Based on the Xiabeishan No.2 tunnel project in China, the mechanical behavior of anchor bolts for shallow super-large-span (SSLS) tunnels in weak rock mass is investigated through laboratory tests, numerical simulation, and field tests. The main conclusions are as follows:

(1) The rock grade significantly affects the bearing characteristics of anchor bolts in SSLS tunnels. In grades IV and V rock mass, the anchor bolts on the side pilot tunnels can always provide significant support to the surrounding rock, while those in the middle pilot tunnel have a limited effect due to the overall subsidence of the overlying rock mass. However, the distribution of the bolt axial force in grade III rock mass is completely opposite to the former, showing a distribution pattern of “large in the arch and small in the sidewall”.

(2) The construction schemes of SSLS tunnels could significantly affect the magnitude and development mode of the bolt axial force, but the final distribution characteristics of the bolt axial force will not change regardless of the construction sequence. Appropriately increasing the bolt length can strengthen the supporting effect of the bolt, but the bolt length should be controlled within 18 m as far as possible to ensure the balance between the supporting effect of the bolt and the engineering economy.

(3) In the field construction, the blasting vibration created by the construction of the middle pilot tunnel caused serious damage to the temporary support, which significantly affected the development of the bolt axial force and caused great construction risks. Therefore, the construction management should be strictly strengthened to guarantee the accuracy of drilling and charging, and to minimize the damage caused by the blasting vibration to the temporary support. In addition, the reinforcement ratio of the temporary support should be appropriately increased to improve the strength and toughness of the temporary support and prevent it from being damaged.

## Figures and Tables

**Table 1:** Support design of the prototype tunnel.

Support Structure		Grade III Rock Mass	Grade IV Rock Mass	Grade V Rock Mass
primary support	C30 shotcrete	thickness: 35 cm	thickness: 45 cm	thickness: 50 cm
	steel frame	I18 steel arch; spacing: 1.2 m	I20b steel arch; spacing: 1.0 m	I22b steel arch; spacing: 0.75 m
	ϕ 32 mm hollow grouting anchor	L = 5 m; array pitch: 2.0 × 1.2 m position: arch and sidewall	L = 6 m; array pitch: 1.5 × 1.0 m position: arch and sidewall	L = 6 m; array pitch: 1.5 × 0.8 m position: arch and sidewall
temporary support	C30 shotcrete	thickness: 25 cm	thickness: 25 cm	thickness: 25 cm
	steel frame	I18 steel arch; spacing: 1.2 m	I20b steel arch; spacing: 1.0 m	I22b steel arch; spacing: 0.8 m
accessory structures	pipe shed	--	--	ϕ 159 mm grouting pipe shed
	lock foot bolt	ϕ 50 mm; t = 4 mm; L = 5 m; position: arch shoulder, arch foot, wall waist, and wall foot		

**Table 2:** Calculation parameters of surrounding rock and support structures.

Type	Thickness(m)	Volume Weight (kN·m^−3^)	Elastic Modulus (GPa)	Poisson’s Ratio	Cohesion (kPa)	Internal Friction Angle (°)
residual slope soil	2	18.5	0.07	0.4	31	28.5
grade V tuff	103.4	20	0.35	0.33	254	9.1
grade IV tuff	111.2	22	0.73	0.30	1372	16.9
grade III tuff	117.4	23	1.72	0.26	2232	24.3
primary support	0.5	25	27.33	0.2	--	--
temporary support	0.25	25	29.65	0.2	--	--
secondary lining	0.7	25	34.9	0.2	--	--

**Table 3:** Calculation parameters of system anchors.

Rock Mass Grade	Anchor Part	Grouting Stiffness (N·m^−2^)	Grouting Cohesion (kPa)	Grouting Friction (°)	Elastic Modulus (GPa)	Grouting Perimeter (m)
III	anchor head	5 × 10^7^	550	50	200	0.314
	anchorage section	5 × 10^6^	55	30	200	0.314
IV	anchor head	2.6 × 10^7^	300	50	200	0.314
	anchorage section	2.6 × 10^6^	30	30	200	0.314
V	anchor head	1.05 × 10^7^	200	50	200	0.314
	anchorage section	1.05 × 10^6^	20	30	200	0.314

Note: the sectional area of the anchor bolt is 3.52 × 10^−4^ m^2^.

**Table 4:** Calculation parameters of pipe sheds.

Type	Sectional Area (m^2^)	Volume Weight (kN·m^−3^)	Elastic Modulus (GPa)	Poisson’s Ratio	Moment of Inertia—z (m^4^)	Moment of Inertia—x (m^4^)	Polar Moment of Inertia (m^3^)
pipe shed	4.68 × 10^−3^	78.5	200	0.27	1.33 × 10^−5^	1.33 × 10^−5^	1.33 × 10^−4^

**Table 5:** Average value and relative error of bolt axial force.

Bolt Axial Force	Section 1				Section 2				Section 3		
	C	D	L	M	C	D	L	M	A	B	N
monitoring (kN)	4.86	8.05	4.68	5.19	4.58	5.64	6.16	4.19	6.88	6.29	6.65
simulation (kN)	4.13	7.96	8.23	4.78	4.11	5.30	6.80	3.81	7.37	6.64	6.67
relative error	15.1%	1.1%	43.2%	8.1%	10.1%	6.0%	9.4%	9.2%	6.6%	5.3%	0.3%

## Data Availability

The data presented in this study are available on request from the corresponding author.
